# Comparison of “framework Shuffling” and “CDR Grafting” in humanization of a PD-1 murine antibody

**DOI:** 10.3389/fimmu.2024.1395854

**Published:** 2024-07-15

**Authors:** Yongmei Wang, Yi-Li Chen, Hui Xu, Gul E. Rana, Xiaorong Tan, Mengying He, Qingqing Jing, Qi Wang, Guifeng Wang, Zuoquan Xie, Chunhe Wang

**Affiliations:** ^1^ Shanghai Institute of Materia Medica, Chinese Academy of Sciences, Shanghai, China; ^2^ University of Chinese Academy of Sciences, Beijing, China; ^3^ Dartsbio Pharmaceuticals Ltd., Zhongshan, Guangdong, China; ^4^ Shanghai Mabstone Biotechnology Ltd., Shanghai, China; ^5^ Antibody Development Department, Shanghai Genechem Co., Ltd., Shanghai, China

**Keywords:** antibody humanization, programmed death-1, FR shuffling, CDR grafting, immunogenicity

## Abstract

**Introduction:**

Humanization is typically adopted to reduce the immunogenicity of murine antibodies generated by hybridoma technology when used in humans.

**Methods:**

Two different strategies of antibody humanization are popularly employed, including “complementarity determining region (CDR) grafting” and “framework (FR) shuffling” to humanize a murine antibody against human programmed death-1 (PD-1), XM PD1. In CDR-grafting humanization, the CDRs of XM PD-1, were grafted into the human FR regions with high homology to the murine FR counterparts, and back mutations of key residues were performed to retain the antigen-binding affinities. While in FR-shuffling humanization, a combinatorial library of the six murine CDRs in-frame of XM PD-1 was constructed to a pool of human germline FRs for high-throughput screening for the most favorable variants. We evaluated many aspects which were important during antibody development of the molecules obtained by the two methods, including antibody purity, thermal stability, binding efficacy, predicted humanness, and immunogenicity, along with T cell epitope prediction for the humanized antibodies.

**Results:**

While the ideal molecule was not achieved through CDR grafting in this particular instance, FR-shuffling proved successful in identifying a suitable candidate. The study highlights FR-shuffling as an effective complementary approach that potentially increases the success rate of antibody humanization. It is particularly noted for its accessibility to those with a biological rather than a computational background.

**Discussion:**

The insights from this comparison are intended to assist other researchers in selecting appropriate humanization strategies for drug development, contributing to broader application and understanding in the field.

## 1 Introduction

Antibody therapeutics have emerged as the fastest growing field of drugs in the world. To date, FDA has approved over 100 monoclonal antibodies (mAbs) (1), extensively utilized in treating conditions such as tumors, viral infections, autoimmune diseases, and organ transplantation (2). Most antibodies were generated through the classic murine hybridoma technology. However, the use of murine antibodies poses high risk of immunogenicity, which diminishes the biological activities and hastens the clearance of therapeutic mAbs by anti-drug antibodies (ADA), and results in side effects in clinical applications (3, 4). Even chimeric antibodies, which retain about 66% of human-like characteristics, have substantial murine sequences (5). Consequently, further humanization is required for most therapeutical applications in human patients. The past three decades have witnessed the evolution of several humanization methods, encompassing CDR grafting, specificity-determining residues (SDR) grafting, resurfacing, framework (FR) shuffling, FR libraries, and guided selection, etc. (6). Two primary trends in the field of humanization include computer- and structure-based rational design, and library-based empirical methods. As a classic method, CDR grafting involves transferring specific antigen-binding CDRs onto human FRs with high homology to murine FRs (7–10). However, it typically results in a substantial reduction in antigen affinity (11, 12), as some key murine residues are crucial for adjusting the conformation of CDRs loop change. The absence of these residues leads to incompatibility between human FRs and non-human CDRs (12–14). Back mutation of these key residues has been typically employed to restore binding affinity (15). CDR grafting relies on computer modeling to identify canonical structure determining residues and design back mutation variants. CDR grafting depends on the precision of computational structural models and the depth of experiential knowledge. If computational predictions are imprecise, or if there is a deficiency in expert knowledge which leads to inadequate selection of critical amino acids for back mutation, the production of an optimal molecule might be compromised. Unlike rational methods that depend on antibody structure or sequence information, FR shuffling is an empirical method relying on constructing and screening large and diverse combinatorial libraries through phage display to select variants with desired properties. This library comprises six CDRs from murine antibodies fused with a pool of diverse human germline FRs, which contains almost all human germline genes of heavy and light chains suitable for antibody humanization (16). The substantial diversity facilitates the selection of optimal human FR combinations that can maintain the dominant conformation of non-human CDRs (17), allowing for sustained high affinities.

In this study, both CDR grafting and FR shuffling were conducted to humanize a chimeric human PD-1 antibody, XM Ch PD-1, with murine CDRs from XM PD-1, a murine antibody. The two strategies were directly compared using evaluation parameters such as production yield, thermal stability, binding activity, blocking efficacy, humanness, and immunogenicity. Notably, the most promising antibody, T5, emerged from the FR-shuffling process. The study highlights FR-shuffling as an effective complementary approach that can potentially increase the success rate of antibody humanization. Although the results are based on a single case study, the dissemination of these comparative insights will hopefully assist other researchers in the pharmaceutical field in selecting effective antibody humanization techniques.

## 2 Materials and methods

### 2.1 Cell lines and reagents

CHO-PD-1 and HEK293F cells were procured from the National Collection of Authenticated Cell Cultures (Shanghai, China). CHO-PD-1 cells were cultured in CD CHO medium (Gibco, New York, USA) supplemented with 0.7 mg/ml geneticin (Gibco, Paisley, UK) and 10 mM glutamine (Gibco, New York, USA). HEK293F cells were cultured in OPM-293 CD05 medium (OPM BiosciencesCo., Ltd., Shanghai, China). Human peripheral blood mononuclear cells (PBMCs) were purchased from MiaoTong Biotechnology (Shanghai, China). E.coli DH10B was a product of Invitrogen(Carlsbad, CA, USA). E.coli XL-1Blue was obtained from Agilent Technologies(USA). M13KO7 helper phage was obtained from NEB(Beijing, China).

PrimeSTAR ^®^ HS DNA Polymerase was from Takara(Beijing, China). T4 DNA ligase and T4 DNA polymerase were purchased from NEB(Beijing, China). Triethylamine was purchased from Sigma(Shanghai, China), and the nitrocellulose filter was purchased from Whatman(Germany). Polyethylenimine (PEI) was purchased from Polysciences(Warrington, PA, USA). Goat anti-human kappa-unlabeled antibodies were procured from Southern Biotech(Birmingham, AL, USA), and PE-labeled goat-anti-human antibodies were purchased from BioLegend(San Diego, CA, USA). Enzymes EcoR I and Nhe I, streptavidin magnetic beads, 1% casein, Immunotube, TMB Substrate Kit, neutravidin-alkaline phosphatase-conjugated, alkaline phosphatase substrate (NBT-BCIP), 5000×SYPRO Orange, and high sensitivity streptavidin-HRP were purchased from Thermo Fisher Scientific. Additional reagents included anti-M13 antibody HRP, PD-1-6xHis, PD-L1-6xHis, GM-CSF, IL-4 TNF-α, IL-1β, IL-6, IL-10 ELISA Kit, IFN-γ ELISA Kit from SinoBiological(Beijing, China). PGE2 was sourced from Absin(Shanghai, China). The EasySep™ Human CD4+ T Cell Isolation Kit was purchased from Stemcell Technologies(Canada), and the CD14 MicroBeads human lyophilized kit was obtained from Miltenyi Biotec(Bergisch Gladbach, Germany).

### 2.2 CDR grafting

The amino acid sequence of murine antibody XM PD-1 was discovered in a previous study (18)and incorporated into the library. Molecular Operating Environment (MOE, version 2019.0102) was utilized for modeling and humanization.

The murine amino acid sequence was loaded into MOE, with annotations made for the regions of FRs and CDRs. The 3D structure model of the antibody was constructed based on FR and CDR templates, respectively. The best mode was selected based on sequence similarity and energy minimization. MOE identified three types of canonical structure determining residues, and the roles of residues in maintaining conformation were confirmed through 3D visualization of the structure. Subsequently, a few human FRs with the highest homology in the Fab sequence database were chosen, and the murine CDRs were grafted onto the selected human FRs. Finally, key residues were designed for back-mutation based on the previously identified canonical structure determining residues and predictions of their effects on structure maintenance and binding ability.

### 2.3 FR shuffling

#### 2.3.1 Construction of the FR shuffling libraries

The process was performed according to the previous literature (16). Oligonucleotides encoding human germline heavy chain FRs served as templates, paired with different primers encoding part of CDRs as homologous arms. The combinatorial library was assembled using overlap extension PCR with biotinylated primers. After capture by streptavidin-labeled magnetic beads, single-strand DNA was obtained by denaturing PCR products in 0.15M NaOH, and the minus single-stranded DNA (non-biotinylated ssDNA) was isolated through 70% EtOH and 3M NAOAc precipitation. The shuffled library, comprising a mixture of light and heavy chains, was annealed simultaneously with the M13 vector containing two palindromic loops. The minus strand was synthesized using T4 DNA ligase and T4 DNA polymerase. The parental template was then digested by *Eco*R I and *Nhe* I restriction enzyme, leaving vectors incorporating both VL and VH. The synthesized DNA was electroporated into DH10B cells for phage packaging (19). A phage solution was prepared through phage amplification in XL-1 Blue cells.

#### 2.3.2 Solution panning

The procedures were carried out as previously reported (19). A total of 5×10^12^ phages were incubated with streptavidin beads for 15 min after being blocked with 1% casein. Subsequently, the solution was pipetted out, and the phages were transferred to a new tube. The bio-PD-1 antigen was then added to the tube and rocked for 1 h. New beads were introduced to pull down the antibody phage-biotinylated antigen complex, and the bound antibody phage was eluted using 100 mM Triethylamine and neutralized with 1 M Tris-HCl at pH 6.4. The XL-1 Blue cells were cultured with the eluted phage for amplification. The phage display library underwent panned for three consecutive rounds.

#### 2.3.3 Immunotube panning

One immunotube was coated with PD-1 antigen protein, and another with phosphate buffered saline (PBS), both standing overnight at 4°C. A total of 5×10^12^ pfu phage was incubated with the PBS immunotube for 1.5 h, rolling up and down, and then standing for an additional 0.5 h for negative selection. Subsequently, the phage was transferred to the immunotube coated with PD-1 antigen, undergoing the same process. The desired phages were eluted with 100mM Triethylamine, and the eluted phages were amplified by infection into XL-1 Blue cells, prepared for the next panning.

#### 2.3.4 Filter lift assay

The procedure was operated following the methods previously reported (19, 20). Phage clones were plated on the bacterial lawn at a density of 2~3×10^3^ pfu plaques per dish. A nitrocellulose filter was overlaid with 5 ml of 2 μg/ml goat anti-human κ antibody-unlabeled for 2 h. Subsequently, the nitrocellulose filter was blocked with 1% casein, air-dried, and applied to the plaque lawn at 22°C overnight. The filter was then exposed to biotinylated PD-1 antigen for 1 h, followed by incubation with neutravidin-alkaline phosphatase for 30 min. Positive clones were ultimately detected using the alkaline phosphatase substrate (NBT-BCIP).

#### 2.3.5 Single point ELISA

50 μl of phage supernatant was incubated with a plate coated with PD-1 antigen for 1 h. Subsequently, the signals were detected using anti-M13 antibody HRP and TMB substrate at OD_450_ nm.

### 2.4 Expression and purification

Regarding the CDR grafting method, the VH and VL sequences were synthesized as designed by Sangon Biotech (shanghai). Conversely, for the FR shuffling method, the VH and VL containing CDRs and shuffled FRs were identified through phage sequencing. The VH and VL regions of the two methods were fused with human IgG1 heavy and light constant domains using DNA recombinant technology. The expression plasmids of heavy chains (HC) and light chains (LC) were co-transfected into HEK293 cells at the ratio of HC: LC=1:3 using polyethylenimine (PEI). The supernatant was harvested after 5-6 days of cell culture, and the antibodies were purified using a protein A column (Smart-lifesciences) and eluted with 0.1M glycine on the AKTA Pure FPLC system (GE Healthcare).

### 2.5 Size-exclusion chromatography

Analytical SEC was performed using the Agilent 1260 HPLC system (Agilent), which was equipped with a Thermo MAbPac SEC-1, 5 µm, 7.8 × 300 mm column (P/N 088460, Thermo Fisher Scientific). A total of 10 µl of sample was injected into the column at a concentration of 1mg/ml. Sodium phosphate (pH 6.8) served as the mobile phase with a flow rate of 1 ml/min. Protein detection was carried out using a UV detector at OD280.

### 2.6 Differential scanning fluorimetry

A working solution of SYPRO Orange 250× was freshly prepared by diluting 1 μl of 5,000×SYPRO Orange in 19μl of water. Subsequently, 1μl of SYPRO Orange working solution was added to 24 μl of PBS-diluted antibodies at a 20 μM. Fluorescence intensities were measured at every 1°C interval within the temperature range of 25 to 95°C using the CF×96 Touch qPCR machine (Bio-Rad). The melting temperature (*Tm*) was calculated from the derivative Relative Fluorescence Unit (RFU) against temperature (dRFU/dT) curve.

### 2.7 Enzyme-linked immunosorbent assay

The 96-well plate (Greiner) was coated with 2 μg/ml PD1-Fc at 4°C overnight, then blocked with 1% casein for 1 h. After washing, three-fold series dilutions from 100nM of antibodies were added to the plates. The color was developed using goat anti human kappa-HRP and TMB substrate at room temperature. After being stopped by 2M H_2_SO_4_, the absorbance was measured at OD_450_ using SpectraMax M5e (Molecular Devices).

### 2.8 Biolayer interferometry

Binding kinetics analysis was conducted on the Octet RED96e system (ForteBio). Protein A biosensors (Sartorius) were pre-wetted in the kinetics buffer (PBS with 0.05% Tween 20) for 10 min. The antibodies were immobilized on the protein A biosensor at a signal level of 1.5 nm. Various concentrations of PD-1-his were applied in a two-fold series dilution in the kinetics buffer (from 100 to 1.56 nM). The association step was set for 120 s, and the disassociation step was set for 180 s. The binding curves were fitted using a global fit 1:1 binding model with ForteBio Data Analysis software 9.0 (ForteBio).

In the self-binding assay, a protein A biosensor captured the antibody to be tested. To ensure specificity, a non-binding antibody blocked any remaining Fc binding sites on the biosensor. The antibody to be tested then underwent a 120s association followed by a 180s dissociation. The signal measured during the association was used to assess the self-binding capabilities of the antibody to be tested, indicating its tendency to interact with itself.

### 2.9 Surface plasmon resonance

The binding kinetics of CDR grafting humanized antibodies were verified by SPR using Biacore T200 (GE Healthcare). The running buffer employed was HBS-EP, consisting of 10 mM HEPES, 150 mM NaCl, 3 mM EDTA at pH 7.4 and 0.005% (v/v) Tween-20. Purified antibodies were diluted to 2 μg/ml and immobilized on Series S Sensor Chip Protein A (GE Healthcare, Cat: 29127556) at 250 response units (RU). The gradient PD-1-ECD-his flowed over the immobilized antibodies starting from 50nM in a two-fold serial dilution, at a flow rate of 30ul/min, with 180 s for association and 1200 s for disassociation. After each cycle, the sensor chip was regenerated with 10mM glycine at pH1.5 for 30 s. The binding kinetics were analyzed using Biacore T200 Evaluation software version 3.1 with a 1:1 Langmuir binding model.

### 2.10 Flow cytometry

Binding of the humanized antibodies to the cell surface PD-1 was measured using Fluorescence-Activated Cell Sorting (FACS). CHO cells overexpressing PD-1 (CHO-PD-1) were aliquoted into FACS tubes at 3×10^5^ cells/tube and then incubated with 2 μg/ml PD-1 antibody at 4°C for 30 min. After washing with PBS twice, the cells were incubated in 100 μl staining buffer containing PE-labeled goat-anti human antibody in the dark for 30 min. The mean fluorescence intensity (MFI) of binding was measured by CytoFLEX flow cytometer (Beckman), and the data were analyzed using FlowJo software (BD Biosciences).

### 2.11 Competitive ELISA

A concentration of 1 μg/ml of recombinant human PD-1-6xHis was coated on 96-well plates (Greiner) at 4°C overnight. Testing antibodies in a 2-fold series dilution were then added to the plates along with 1 μg/ml biotinylated PD-L1. Following a 1 h incubation, the bound ligand was detected using streptavidin-HRP and developed with TMB substrate. The reaction was halted by 2M H_2_SO_4_, and the absorbance was measured using SpectraMax M5e (Molecular Devices).

### 2.12 Mixed lymphocyte reaction assays

CD14^+^ and CD4^+^ cells were isolated from frozen PBMCs from different donors using the CD14 MicroBeads human lyophilized kit and EasySep™ Human CD4^+^ T Cell Isolation Kit. The isolated CD14^+^ cells were cultured with 500 ng/ml GM-CSF and 500 ng/ml IL-4 for 5 days to generate immature DCs. Subsequently, they were treated with 30 ng/ml TNF-α, 300 ng/ml IL-1β, 300 ng/ml IL-6, and 3 μg/ml PGE2 for an additional two days to induce DC maturation. On day 8, CD4^+^ T cells (1×10^5^ cells) were co-cultured with allogeneic DCs (1×10^4^ cells) in the presence of T5, Pembrolizumab, Nivolumab, and isotype. After 5 days of culture, the supernatant was collected to measure the production of IL-10 and INF-γ using ELISA.

### 2.13 Prediction of humanness and immunogenicity

BioPhi was a platform designed for antibody design, humanization, and humanness evaluation, which leveraged natural antibody repertoires and deep learning technologies (21). It incorporated a component called OASis, which provided a detailed, interpretable humanness score. The sequences of the light chain and heavy chain were inputted on this website https://biophi.dichlab.org, and the OASis score was outputted.

T20 humanness analyzer was another classic prediction tool (22). The sequences of the light and heavy chains were inputted on this website http://abAnalyzer.lakepharma.com, and the T20 score was outputted.

### 2.14 Molecular dynamics simulations

The structures of the murine antibody was obtained by homology modeling by MOE. The antibodies were annotated according to Kabat. The antibody Fv was solvated in a cubic water box that is sufficiently large to provide a minimum buffer zone of 14 Å between biological material and the cubic system boundaries. Na^+^ and Cl^-^ ions were randomly placed to neutralize the system electrostatically at a physiological salt concentration of 0.150 M. The CHARMM36m force field (23) and the three-site OPC water model were used subject to periodic boundary conditions (24). The initial energy minimization was performed using the steepest descent method. Later, the system was equilibrated in the NVT ensemble at 310 K for 100 ps, using a small integration time step of 2 fs. Production trajectories were recorded in the NPT ensemble at 310 K and 1 atm atmospheric pressure using a 2 fs of integration time steps for a total of 100 ns. Atomic coordinates were saved every 10 ps. The long simulation utilized similar parameter settings, with a total duration of 1 μs. Gromacs 2024.1 was used for simulation setups and trajectory collection. Gromacs and in-house Python scripts were used for all analyses.

### 2.15 Comparative analysis of residual interactions across predicted protein structures

We developed a comprehensive method to analyze protein-protein interactions within a structure, utilizing the Bio.PDB module from the BioPython library. Our method focuses on identifying four primary types of molecular interactions critical to protein function and stability:

Hydrogen Bonds: Analyzed between nitrogen (N) and oxygen (O) atoms within a 3.5 Å threshold.

Hydrophobic Contacts: Evaluated between side-chain carbons of hydrophobic residues (Leucine, Isoleucine, Valine, Phenylalanine, Tryptophan, Methionine) excluding alpha carbons, with a distance limit of 5.0 Å.

Salt Bridges: Assessed between oppositely charged residues (Arginine, Lysine, Histidine; Aspartic acid, Glutamic acid) within a 4.0 Å range.

Pi-Pi Stacking: Investigated between aromatic rings (Phenylalanine, Tyrosine, Tryptophan, Histidine) up to 6.5 Å apart.

### 2.16 Statistical analysis

Statistical evaluation was conducted using GraphPad Prism 8.0 (GraphPad Software Inc.). P values were calculated using a one-way ANOVA multiple comparison test (*P < 0.05, **P < 0.01, ***P < 0.001, ns: not significant).

## 3 Results

### 3.1 Generation, screening and sequence analysis of humanized antibodies

The design of CDR-grafting humanization was conducted using software Molecular Operating Environment (MOE) (25). The annotation of antibodies was performed using the IMGT(The International ImMunoGeneTics information system). The murine antibody structure model was built based on the FR and CDR templates retrieved from the PDB database. The templates and corresponding similarity scores were shown in **Supplementary Table 1**. Three types of canonical structure determining residues were identified through the predicted 3D structure (**Supplementary Tables 2**, **3**). Type 1 residues, located at the binding interface between VL and VH, play key roles in the packing of the two structural domains. Type 2 residues, positioned close to the CDR region and embedded in the protein interior, may affect the overall antigen-antibody binding conformation. Type 3 residues have direct interactions with the CDR region, including hydrophobic interactions, hydrogen bonds, and salt bridges. The robustness of our MOE template modeling has been validated through comparative analyses with models generated by ABodyBuilder (26) and ImmuneBuilder (27). The results, as shown in **Supplementary Figure 1**, indicate that the overall structural frameworks were consistent across the models, with RMSD values of 0.48 Å between MOE and ABodyBuilder, and 0.393 Å between MOE and ImmuneBuilder. This consistency underscores the reliability of the MOE model, especially within the framework regions (FR).The RMSDs calculated separately for the CDR loops and the remaining Fv regions were shown in **Supplementary Table 4**). Static modeling provides a single, often idealized snapshot of the molecular structure, without accounting for the natural fluctuations and movements that molecules undergo in their native environments. Molecular Dynamics (MD) simulation captures the time-dependent behavior of molecules and incorporates environmental effects, enabling researchers to observe how structures and interactions evolve over time. **Supplementary Figure 2A** displayed the Root Mean Square Deviation (RMSD) values for XM Ch PD1 in MD simulations (100 ns), showing fluctuations between 6.5 and 12.5 ns, yet not exceeding 0.23 nm. After approximately 15 ns of MD simulation, the system began to stabilize, ultimately settling at 0.09-0.17 nm. This suggested that the simulated structure closely matched the predicted structure from homology modeling. Additionally, long simulations (1 µs) were employed to gain deeper insights into the dynamic behavior of conformation-stabilizing contacts and to potentially correct minor inaccuracies in the modeled structures. The RMSD values, ranging from 0.069 to 0.258 nm (**Supplementary Figure 2B**), indicated that the antibody maintained a relatively stable structure with some conformational flexibility over the 1 μs MD simulation. This further indicated that the simulation conditions were appropriately set, and the initial model was reliable, without resulting in significant structural changes.

The conventional CDR-grafting method employs a single human germline sequence for grafting parent CDRs, often failing to satisfy all key requirements: low immunogenicity, high stability, and high expression. To address this, each non-human FR was individually humanized by selecting the most homologous human germline FR, allowing the preservation of the original antibody’s properties to the greatest extent. The chosen germlines of CDR grafting were shown in **Table 1**. After grafting the CDRs onto human FRs, the designed molecule was named ‘human-germline-grafted’. By comparing the designed one and the murine antibody XM Ch PD-1, the residues different in the FR region were identified. Three types of canonical structure determining residues listed by the MOE were only for reference, we determined the key residues by comprehensive considerations. To determine which key residues need back-mutation to retain the binding ability, their roles in conformation maintenance were checked using the constructed parental structure model. The sites involved in the mutations include K19, T40, E42, R44, P61, A63, V70, E76, E77, I78, K87, and S88 in the heavy chain and the sites involved mutations include M37, H38, and P47 in the light chain(**Supplementary Figure 3**). Finally, eight humanized heavy chains and four humanized light chains were designed. We generated 32 humanized antibodies. Through single point ELISA screening, four candidates with the highest binding, named BS#3, BS#5, BS#11, BS#31 were selected for subsequent studies.

**Table 1:** The germlines used for CDR-grafting humanization.

	FR1	FR2	FR3	FR4
VH	IGHV3-15*01	IGHV3-7*01	IGHV3-74*01	JH5
VL	IGKV3D-11*02	IGKV3-11*01	IGKV6-21*01	JK4

**Figure 1:**
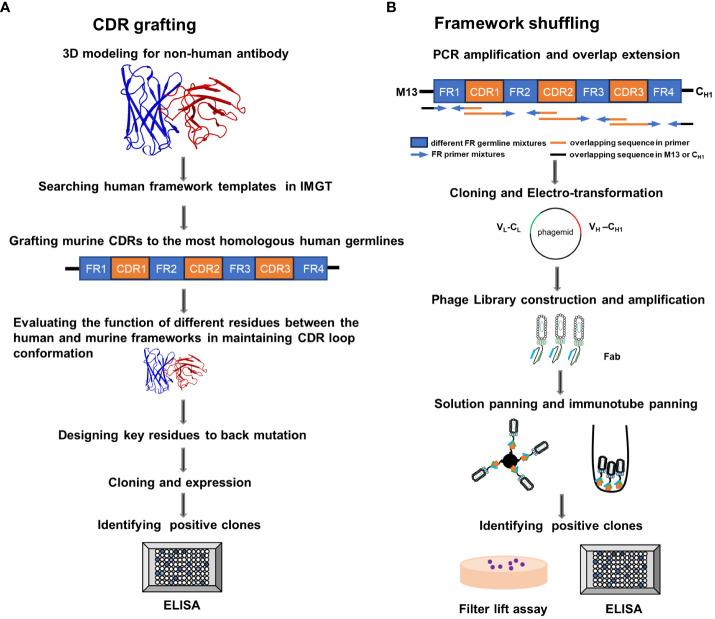
Schematic illustration of the complementarity determining region (CDR) grafting and framework (FR) shuffling humanization strategies. **(A)** The main procedure of the CDR grafting. **(B)** The main process of the FR shuffling.IMGT, the international ImMunoGeneTics database; ELISA, enzyme-linked immunosorbent assay; PCR, polymerase chain reaction; Fab, fragment of antigen binding. V_L_, Light chain variable region; C_L_, light chain constant region; V_H_, heavy chain variable region , C_H1_, heavy chain constant region 1.

FR-shuffling humanization was executed by selecting different human heavy and light (κ) germline genes into each of the four FRs while keeping the CDRs fixed (16). The full-length variable structural domains were generated by fusing CDRs with the FR pool. The gene diversity of the library exceeded 4×10^6^, which was ample for selecting the optimal matches between CDRs and FRs. After multiple rounds of solution panning and immunotube panning, the capture lift assay was developed for the initial screening of the large library (19, 20). Only deep colored spots were isolated as positive clones. Single point ELISA was employed for the secondary screening. The schematic diagrams of the FR shuffling and CDR grafting methods are shown in **Figure 1**. We selected the top nine candidate molecules with the highest OD values from a single-point ELISA binding assay, representing the molecules with the best binding affinity, named T1-T9.

The humanized heavy and light chains were completely shuffled as they derived from various human germline families. For example, T1 Ab contained VH5/VH3 and VK6/VK3, respectively (**Table 2**). With a more nuanced insight, three FRs of individual heavy and light chains may derive from different human germline genes with in a given family, such as T5 Ab compromised VH3-15 and VH3-53 in the heavy chain. Overall, the humanized PD-1 antibodies’ heavy chain showed a preference for the VH3 germline family. This inclination could be attributed to the greater homology of this class with the parental FR, thereby increasing the likelihood of preserving essential parental residues (16). Besides, various germline families were exhibited in the humanized light chain.

**Table 2:** Sequence analysis of FR-shuffled antibodies.

mAbs	Human VH germline				Human VL germline			
	FR1	FR2	FR3	FR4	FR1	FR2	FR3	FR4
T1	IGHV5-51	IGHV3-15	IGHV3-23	JH5	IGKV6-21	IGKV3-15	IGKV6-21	JK4
T2	IGHV3-15	IGHV1-46	IGHV1-46	JH5	IGKV1-5	IGKV3-15	IGKV2-28	JK4
T3	IGHV3-23	IGHV1-46	IGHV3-53	JH5	IGKV5-2	IGKV3-15	IGKV4-1	JK4
T4	IGHV3-23	IGHV3-15	IGHV3-72	JH5	IGKV5-2	IGKV4-1	IGKV3-15	JK4
T5	IGHV3-15	IGHV3-15	IGHV3-53	JH5	IGKV5-2	IGKV3-15	IGKV2-28	JK4
T6	IGHV3-72	NA	IGHV3-72	JH5	IGKV5-2	IGKV3-15	IGKV4-1	JK4
T7	IGHV3-23	IGHV3-72	IGHV3-72	JH5	IGKV5-2	IGKV3-15	IGKV4-1	JK4
T8	IGHV3-53	IGHV4-4	IGHV3-72	JH5	IGKV1D-43	IGKV3-15	IGKV6-21	JK4
T9	IGHV3-15	NA	IGHV3-53	JH5	IGKV1-5	IGKV3-15	IGKV4-1	JK4

FR, framework; NA, not applicable, for the mutations in the region.

### 3.2 FR shuffling-humanized antibodies showed higher titers and purities

**Figure 2:**
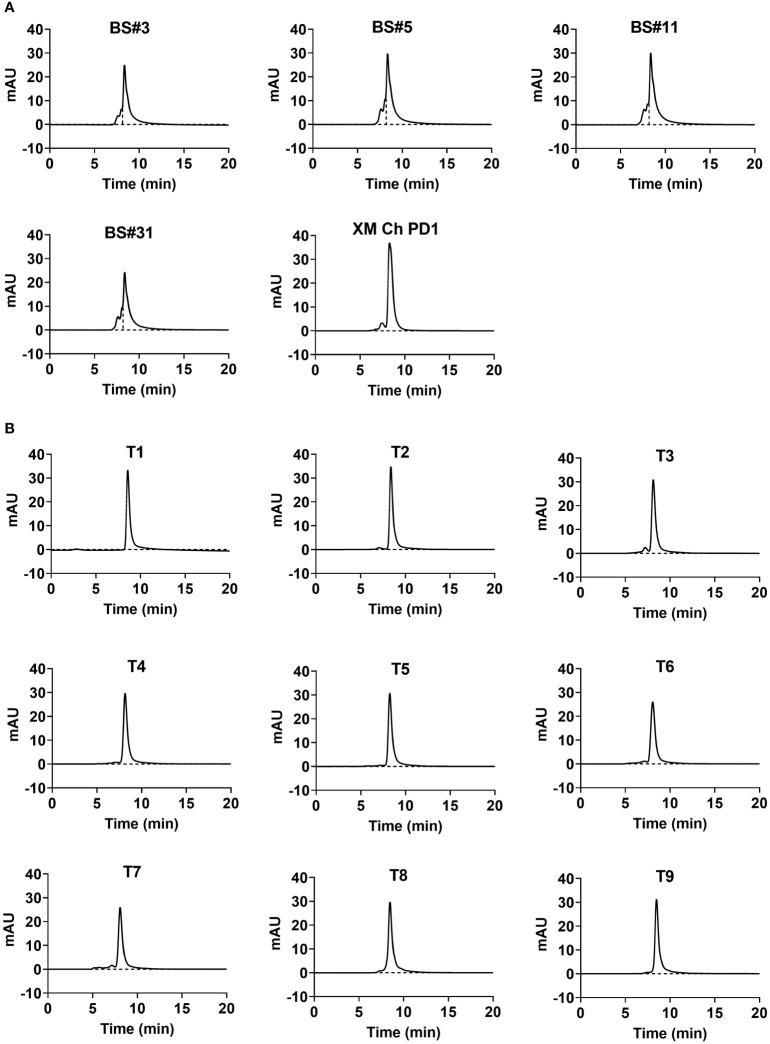
The aggregation and purity of humanized antibodies. The purity of humanized variants generated by **(A)** CDR grafting and **(B)** FR shuffling detected by size-exclusion chromatography (SEC).

The top mammalian expression titers of selected CDR grafting antibodies (BS# series) were approximately 100 mg/L, and most of the FR shuffling-humanized antibodies (T series) yielded titers higher than 100 mg/L, except T6 and T8 (**Table 3**). Size exclusion chromatography (SEC) were used to assess the purities of antibodies after a single-step purification with Protein A columns. Peaks that elute faster in the chromatogram represented larger aggregates. All the CDR grafting-humanized antibodies aggregated to an extent of about 20% (**Figure 2A**). In comparison, all nine FR shuffling-humanized antibodies exhibited minimal aggregation with >90% purity as revealed by analytical SEC (**Figure 2B**). Thus, T1-5, T7 and T9 were selected for further studies. For subsequent head-to-head comparison, we used SEC for secondary purification to remove aggregates and improved the antibody purities to nearly 100%. Once the aggregates were removed, the CDR grafting-humanized remained stable in the PBS buffer.

**Table 3:** Production yields of humanized PD-1 antibodies in HEK 293 cells.

Antibodies	Production (mg/L)
BS#3	97
BS#5	117
BS#11	101
BS#31	99
T1	172
T2	160
T3	150
T4	133
T5	159
T6	86
T7	156
T8	28
T9	114

### 3.3 FR shuffling-humanized antibodies exhibited better thermal stability

**Figure 3:**
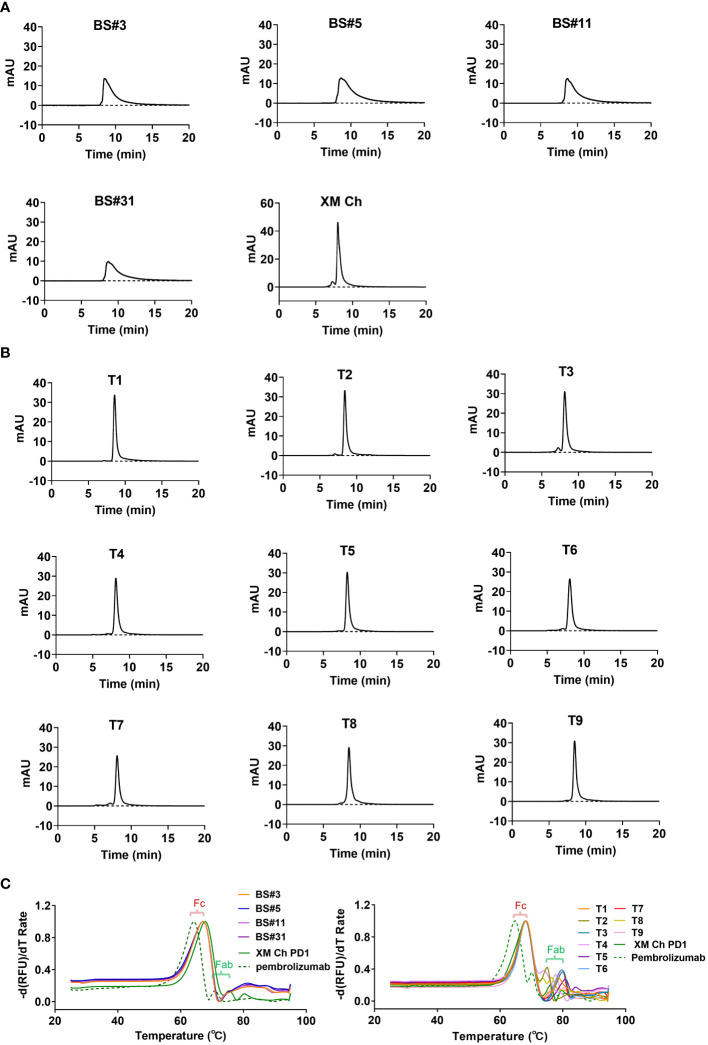
The thermal stabilities of the antibodies obtained from the two different humanization methods. **(A, B)** The detection of degradation using SEC after heating at 60°C for one hour for CDR grafting- and FR shuffling-humanized antibodies. **(C)** Differential scanning fluorimetry (DSF) was used to determine the melting temperature (*Tm*) values of proteins.

After being heated at 60°C for one hour, the purity of the humanized antibodies was analyzed by SEC to determine their thermal stabilities. Protein peaks that elute after the main peak were typically degraded smaller fragments, which traveled through the gel matrix slower than the intact antibody molecules. The four candidates obtained by CDR grafting were not thermally stable, and degraded more when compared to the chimeric antibody XM Ch PD-1 (**Figure 3A**). In contrast, the nine candidates obtained by FR shuffling were thermally stable after being heated (**Figure 3B**).

Additionally, Differential Scanning Fluorimetry (DSF) was used to measure the thermal stability of the antibodies. Using extrinsic fluorescent probes and real-time PCR apparatus, DSF can monitor unfolding transition of specific proteins with increasing temperature (28). As shown in **Figure 3C**, all the *Tm* values of the antibodies’ Fc domains were at 68.2°C, consistent with the chimeric antibody. The melting temperatures were shown in **Supplementary Table 5**. The Fab domains of T1, T3, T5 and T6-9 exhibited enhanced thermal stability compared with its murine counterpart XM Ch PD-1, and the *Tm* values of the antibodies were all above 73.9°C. Thus, the selected FRs and CDRs are compatible to each other. In contrast, the *Tm* values of the Fabs of the four CDR grafting-generated variants were under 68.2°C (**Figure 3C**). Thus, they are inferior to FR shuffling-generated variants generated in thermal stability.

### 3.4 FR shuffling-humanized antibodies retained binding affinities

**Figure 4:**
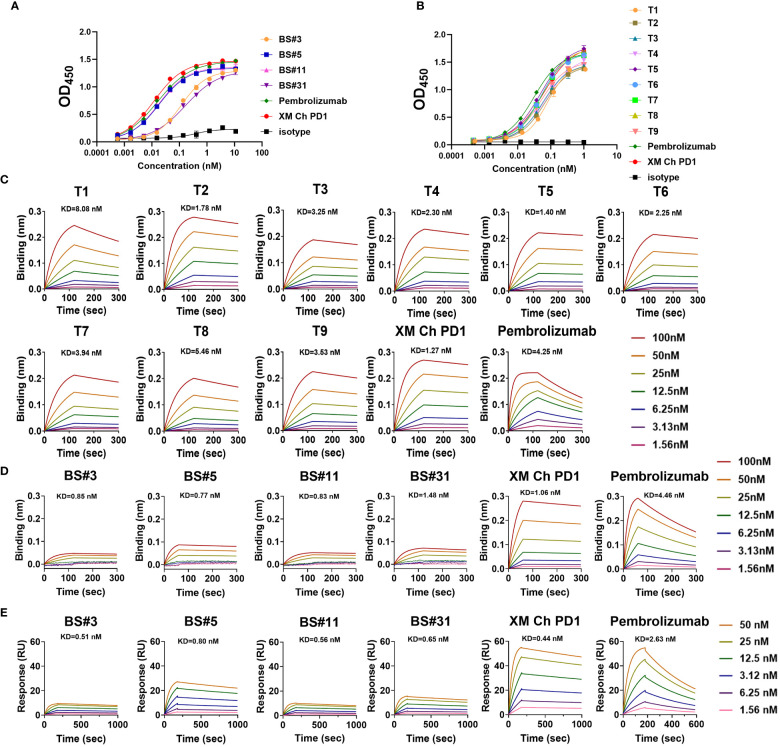
The binding abilities of humanized antibodies. **(A, B)** The binding affinity for CDR grafting variants and FR shuffling variants with PD-1 protein measured by enzyme-linked immunosorbent assay (ELISA), respectively. **(C)** The binding kinetics of FR shuffling variants detected by biolayer interferometry (BLI). **(D, E)** The low binding response of CDR grafting variants verified by BLI and surface plasmon resonance (SPR). The positive control pembrolizumab was approved by the FDA in 2014 for use in unresectable or metastatic solid tumors.

The binding affinities of humanized antibodies were compared by ELISA. The two CDR grafting-humanized variant BS#5 and BS#11, and the nine FR shuffling-humanized variants all showed high affinities similar to that of the chimeric antibody (**Figures 4A, B**).

The binding kinetics was evaluated by biolayer interferometry (BLI), which monitors the rates of association and disassociation in real-time with precision and accuracy (29). The nine FR shuffling-humanized variants showed high binding affinities in BLI (**Figure 4C**), with T5 as the highest affinity antibody. In contrast, CDR grafting-humanized variant BS#3, BS#5, BS#11 and BS#31 exhibited much lower binding affinities than XM Ch PD-1 and the positive control pembrolizumab. Surface plasmon resonance (SPR) is another reliable label-free detection method for biomolecular interactions (30), which showed similar results to BLI (**Figures 4D, E**).

**Figure 5:**
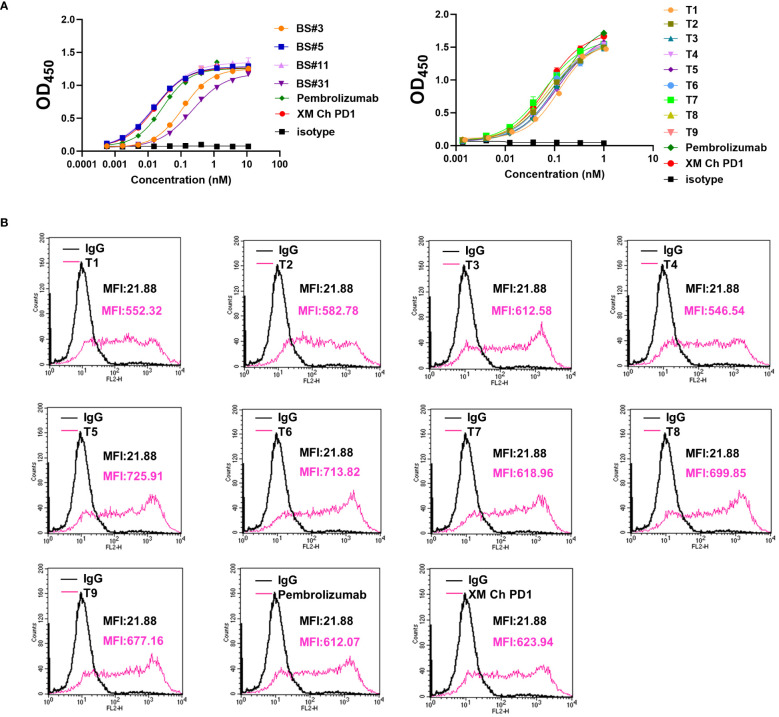
The binding properties of FR shuffling antibodies were further analyzed. **(A)** The binding activities of humanized antibodies after heating to PD-1 protein. **(B)** The binding activities of humanized antibodies to PD-1 on the CHO cells. PE-labeled anti-human IgG was used, and the mean fluorescence intensity (MFI) of binding was measured by flow cytometry.

Next, we compared the binding activities of the variants after being heated, which demonstrated that FR shuffling-humanized variants were superior to CDR grafting-humanized ones (**Figure 5A**).

Based on the results above, we focused on developing molecules of FR shuffling. The nine FR-shuffling variants were assayed for binding abilities to PD-1 overexpressed on CHO cells. They showed comparable binding activities to XM Ch PD-1 and pembrolizumab, with T5 as the highest-binding variant (**Figure 5B**).

### 3.5 Humanized antibodies blocked PD-1/PD-L1 interaction

**Figure 6:**
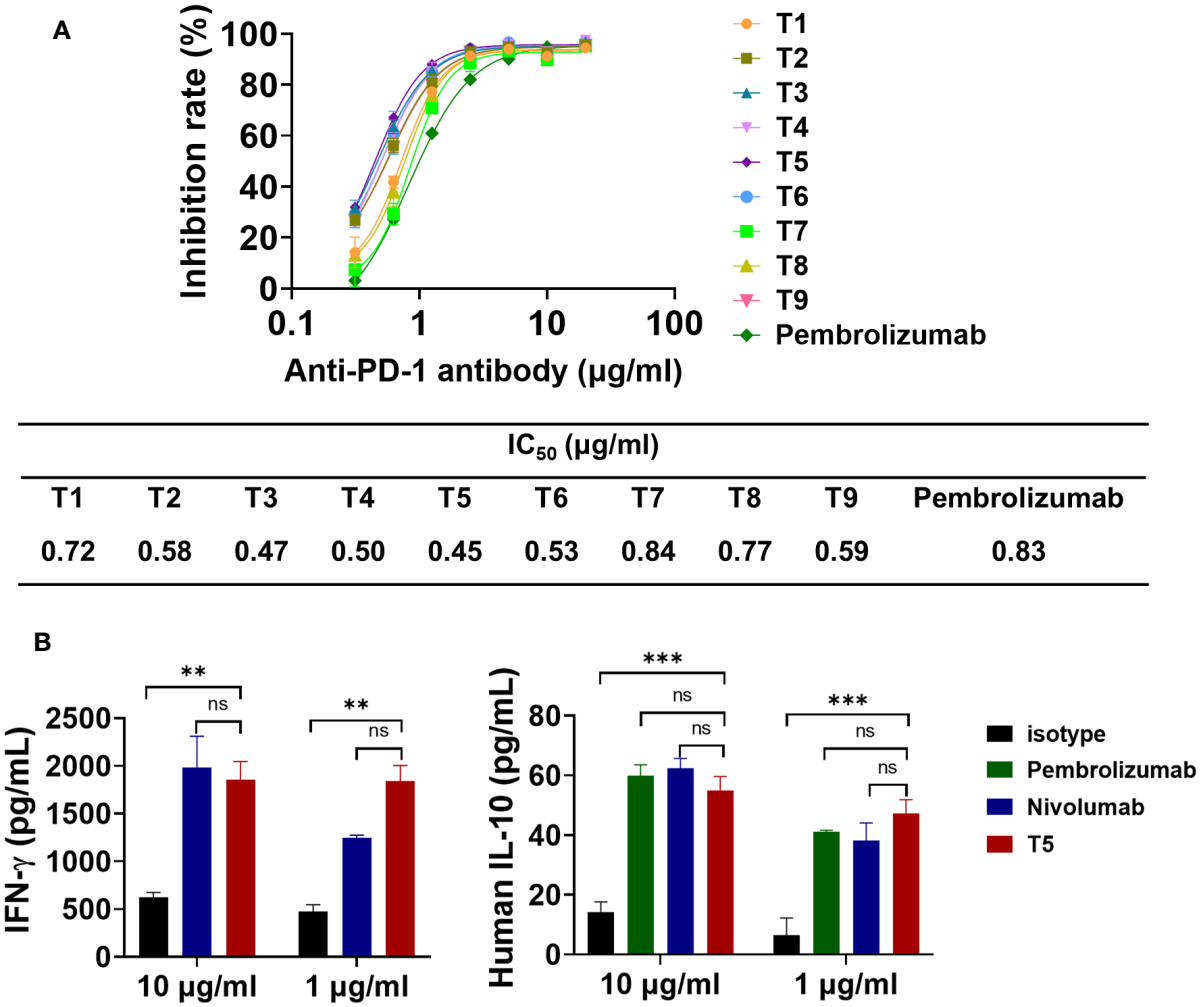
The blocking effects of FR shuffling antibodies on PD-1/PD-L1 interaction. **(A)** The humanized variants of FR shuffling suppressed PD-1/PD-L1 interaction by competitive ELISA. Data are presented as means ± SEM of triple replicates. **(B)** The promising candidate T5 activated T cell responses and induced INF-γ and IL-10 secretion in mixed lymphocyte reaction(MLR) assays. Nivolumab (Opdivo^®^) was one of the first PD-1 antibodies to receive FDA approval. The data were presented as mean ± SEM and were analyzed by one-way ANOVA.  ***P < 0.001, **P < 0.01 were regarded as statistically significant and ns as not significant.

The effects of the FR shuffling-humanized variants on the interaction between PD-1 and PD-L1, were measured (31). All nine variants could effectively block the binding between PD-1 and PD-L1. The *IC_50_* values of the variants ranged from 0.45 to 0.84 nM, slightly better than pembrolizumab. Among them, T5 was the best variants with *IC_50_* values of 0.45 nM (**Figure 6A**). In addition, the functional activity of FR-shuffling variant T5 was analyzed by MLR assay (32). As shown in **Figure 6B**, T5 significantly stimulated IL-10 and INF-γ production at both 1 and 10 μg/ml, which was comparable to positive control pembrolizumab and nivolumab.

### 3.6 Prediction of humanness and immunogenicity for humanized antibodies

We used two different methods to predict the humanness of the humanized antibodies, BioPhi and T20 humanness analyzer, as shown in **Table 4**. The results showed that the variants from both humanization strategies were similar in degrees of humanness and all had improved humanness compared to the murine counterpart. FR-shuffling variant T5 was one of the variants with the highest humanness.

**Table 4:** Humanness prediction *in silico* by OASis Identity and T20.

Antibodies	OASis Identity			T20	
	whole antibody	VH	VL	VH	VL
XM Ch PD-1	59.15%	60.36%	57.84%	69.96%	65.23%
T1	71.36%	73.87%	68.63%	72.82%	75.63%
T2	70.89%	72.97%	68.63%	70.97%	70.68%
T3	71.83%	75.68%	67.65%	82.56%	67.61%
T4	71.83%	74.77%	68.63%	78.61%	68.02%
T5	72.30%	76.58%	67.65%	81.39%	65.95%
T6	69.48%	73.87%	64.71%	77.52%	67.61%
T7	72.30%	76.58%	67.65%	80.84%	67.61%
T8	70.42%	75.68%	64.71%	78.49%	69.73%
T9	72.30%	74.77%	69.61%	82.06%	74.32%
BS3#	72.30%	71.17%	73.53%	75.34%	78.33%
BS5#	69.95%	71.17%	68.63%	75.34%	75.77%
BS11#	70.89%	71.17%	70.59%	75.34%	76.53%
BS31#	72.30%	71.17%	73.53%	75.34%	77.39%

Analyzing the potential T cell epitopes has been suggested to provide valuable forecasts for immunogenicity estimation. Thus, netMHCIIpan (4.0) was implemented to predict the potential peptides binding to HLA II. Due to the extensive polymorphism of HLA II in the general population, which presents a formidable obstacle in T cell epitope identification, we chose the most frequent alleles in loci DRB1 to represent the coverage of HLA II in the population (33, 34). As shown in **Table 5**, T5 demonstrated a reduction in affinity for HLA II alleles when compared to its murine counterpart, suggesting a decrease in potential immunogenicity.

**Table 5:** The potential immunogenicity by predicting the number of strong binders to HLA II alleles.

Alleles	XM PD-1 VH	T5 VH	XM PD-1 VL	T5 VL
DRB1*0101	1	1	0	0
DRB1*0301	1	0	0	0
DRB1*0401	2	1	1	1
DRB1*0405	2	1	2	1
DRB1*0701	0	0	0	0
DRB1*0901	0	0	0	0
DRB1*1101	1	1	0	0
DRB1*1201	0	0	0	0
DRB1*1302	0	0	1	1
DRB1*1501	0	0	1	1
total	7	4	5	4

The HLA II alleles were selected as the most frequent in the general worldwide population according to the literature (34). Each allele has a different frequency in population. The threshold for a strong binder was set at 1% rank in default.

## 4 Discussion

For murine antibodies generated using hybridoma technology, humanization is typically necessary before clinical applications to minimize potential immunogenicity in human. This study compared two popular strategies of antibody humanization in the industry: CDR grafting and FR shuffling.

The comparative analysis of two humanization methods was applied to a PD-1 antibody, revealing distinct advantages of FR shuffling over CDR grafting. CDR grafting-humanized variants exhibited a higher propensity for aggregation (nearly 20%), whereas FR shuffling-humanized variants demonstrated higher purity post-purification. Antibody aggregation reduces biological activity and may elicit immunological responses *in vivo* (35–37). The initial purification step involved Protein A column, which did not effectively remove aggregates. Subsequent size exclusion chromatography (SEC) was employed as a secondary purification step, successfully eliminating the aggregates. The cessation of antibody aggregation following the second purification indicates that most aggregation likely occurred during cell culture or the binding and elution process on Protein A, where antibodies were exposed to physical stress, pH fluctuations, and changes in ionic strength, potentially destabilizing their structure and leading to aggregation. Despite these challenges, the antibody T series exhibited superior structural and conformational stability, maintaining structural integrity under such stressful conditions.

Furthermore, the CDR grafting-humanized variants exhibited poor thermal stability, leading to significant antibody degradation upon heating. Additionally, these variants demonstrated markedly lower binding responses in BLI and SPR assays. In contrast, the FR shuffling-humanized variants exhibited robust binding responses and higher affinities compared to both the chimeric version and pembrolizumab. The discrepancy in antigen-antibody binding signals observed between ELISA and binding kinetics assays indicates more efficient and stable binding of FR shuffling-humanized variants relative to CDR grafting-humanized ones. This discrepancy in maximum binding between ELISA and kinetics assays can be attributed to several factors. Firstly, ELISA employs enzymatic amplification to enhance assay sensitivity, facilitating detection of even low-affinity interactions. In contrast, BLI and SPR directly measure changes in interference pattern or refractive index due to binding events without amplification, potentially rendering them less sensitive to weak interactions. Secondly, ELISA, being an end-point assay, quantifies the total amount of bound antigen after a specific incubation period, which can capture slow or weak interactions. In contrast, BLI and SPR measure binding kinetics in real-time, allowing differentiation of signals for weak or fast-dissociating interactions. Importantly, it was confirmed that there was no self-binding between the variant molecules, thereby excluding self-binding as a cause for the observed performance differences (**Supplementary Figure 4**).

The stability of the antibodies was further assessed using DSF. The *Tm* values of the CDR grafting-humanized antibodies were found to be below 68.5°C, whereas those of the FR shuffling-humanized variants exceeded 73°C. A higher *Tm* value indicates greater thermal stability, indicative of a well-packed structure that requires more energy to unfold and is less likely to undergo aggregation (38). Noteworthy, all nine variants generated through FR shuffling retained their binding ability even after undergoing accelerated thermal treatment. The lower thermal stability observed in CDR grafting-humanized antibodies may lead to increased aggregation propensity, susceptibility to degradation, and challenges in manufacturing, shipping, and storage. These factors can contribute to delays and escalate costs in drug development processes.

After humanization, humanness was evaluated for immunogenicity prediction. BioPhi was a platform designed for antibody design, humanization, and humanness evaluation, which leveraged natural antibody repertoires and deep learning technologies. The OASis score was calculated based on a search of 9-mer peptides within the Observed Antibody Space (OAS) database, ensuring a diverse and granular evaluation of antibody sequences for their similarity to natural human antibodies (21). The T20 humanization assessment tool was developed based on an in-depth analysis of the humanness of therapeutic antibodies using a large number of human antibody sequences from the NCBI Igblast database. It can efficiently distinguish human sequences from non-human sequences (22). As expected, both CDR grafting and FR shuffling improved the humanness of XM Ch PD-1. Besides, T5 exhibited superior biophysical and biological features in comprehensive consideration.

The netMHCII 4.0 tool was trained on natural ligands eluted from HLA II by mass spectrometry and binding affinity to HLA II, a state-of-the-art predictor tool for the analysis of T cell epitopes considering biological features in the process (39, 40). It seemed that T5 decreased the number of T cell epitopes compared with the murine counterpart. However, given the MHC-II polymorphism and diverse allotype tolerance of patients in clinic, it’s still a great challenge to predict the immunogenicity of therapeutic antibodies in the field.

The orientation of the heavy and light chain in the interface is heavily influenced by crystal packing effects. The templates for the homology model are based on crystal structures. This factor sometimes compromises the reliability of residues identified as type 1 canonical structure determining residues. To address this issue, we compared the residues and their interactions across three predictive models: MOE, ABodyBuilder, and ImmuneBuilder(**Supplementary Tables 6** and **7**). Importantly, discrepancies observed among these models did not involve mutated residues. Moreover, interactions identified across the different models exhibited a high degree of similarity, underscoring the robustness and consistency of our modeling approach. MD simulations capture the evolution of molecular structures and interactions over time, providing insights into processes like protein folding and conformational changes. Integrating MD simulations with traditional homology modeling can significantly enhance the precision and efficacy of antibody engineering and development process.

The CDR grafting dataset is characterized by a fixed combination of germlines, whereas the framework shuffling dataset exhibits considerably more diversity. To optimize the CDR grafting process, it is advisable to consider incorporating the top three germlines instead of solely relying on the single best match, unless there is a significant decrease in homology. By employing multiple germline datasets in CDR grafting, there is potential to achieve enhanced results. This approach facilitates a broader exploration of germline diversity, which can improve the efficacy of the humanization process. It also allows for a more comprehensive evaluation of candidate molecules, potentially leading to better outcomes in terms of antibody stability, affinity, and other critical properties. Incorporating multiple germline datasets thus represents a strategic enhancement in optimizing CDR grafting for antibody engineering and therapeutic development.

Both CDR grafting and FR shuffling stand as pivotal methodologies in the humanization of antibodies. While CDR grafting holds a historical precedence in antibody humanization, its efficacy can be hindered by the intricate task of identifying suitable residues for back mutations, a process reliant on precise structural modeling. Moreover, the iterative nature of designing and evaluating variants in CDR grafting can prolong the humanization process. Furthermore, CDR grafting is inherently personalized, with back mutations varying from one antibody to another, thus placing a heavy emphasis on the expertise and experience of the designer. In contrast, the adoption of the FR shuffling method offers a promising avenue to enhance the success rate of antibody humanization. Through the utilization of a highly diverse FR shuffling library, optimal combinations of CDRs and FRs can be selected, thereby increasing the likelihood of retaining affinities. Notably, the comprehensive nature of the FR library, encompassing a wide array of human heavy and light chain germline genes, renders this method applicable across various antibody targets. Illustratively, our laboratory has successfully employed the FR shuffling method in the humanization of antibodies targeting PD-L1 (41) and TIGIT (42), among others. These humanized antibodies have demonstrated satisfactory thermal stabilities, affinities, and biological functionalities underscoring the effectiveness of the FR shuffling approach in antibody engineering.

FR shuffling is particularly accessible to those with a biological rather than computational background, contributing to the growing popularity of humanized techniques in the industry. The insights derived from this comparison aim to assist researchers in selecting suitable humanization strategies for drug development, thereby fostering broader application and a deeper understanding of this field.

## Data availability statement

The raw data supporting the conclusions of this article will be made available by the authors, without undue reservation.

## Ethics statement

Ethical approval was not required for the studies on humans in accordance with the local legislation and institutional requirements because only commercially available established cell lines were used. The study was conducted in accordance with the local legislation and institutional requirements.

## Author contributions

YW: Data curation, Formal analysis, Investigation, Methodology, Software, Validation, Visualization, Writing – original draft, Writing – review & editing. Y-LC: Data curation, Formal analysis, Methodology, Software, Validation, Visualization, Writing – review & editing. HX: Data curation, Formal analysis, Methodology, Software, Validation, Visualization, Writing – review & editing. GR: Formal analysis, Visualization, Writing – original draft, Writing – review & editing. XT: Data curation, Formal analysis, Methodology, Software, Validation, Visualization, Writing – review & editing. ZX: Conceptualization, Funding acquisition, Resources, Supervision, Writing – review & editing. MH: Data curation, Formal analysis, Investigation, Methodology, Validation, Visualization, Writing – review & editing. QJ: Data curation, Formal analysis, Methodology, Software, Writing – review & editing. QW: Software, Conceptualization, Funding acquisition, Investigation, Project administration, Resources, Supervision, Writing – review & editing. GW: Formal analysis, Investigation, Writing – original draft, Conceptualization, Funding acquisition, Project administration, Resources, Supervision, Visualization, Writing – review & editing. CW: Funding acquisition, Project administration, Resources, Supervision, Visualization, Writing – review & editing, Conceptualization.
