# Supplementary material for: Comparison of “framework Shuffling” and “CDR Grafting” in humanization of a PD-1 murine antibody
Source: Front Immunol. 2024 Jul 15;15:1395854. doi: 10.3389/fimmu.2024.1395854 (PMC11284016; doi:10.3389/fimmu.2024.1395854)
Supplement: Supplementary file 1 [file DataSheet_1.docx]

**Supplementary Table 1.** Selected templates and sequence similarity for each domain

| **Domain** | **Template PDB ID** | **Similarity score** |
| --- | --- | --- |
| FR L for murine Ab | 4U1G | 91.7 |
| FR H for murine Ab | 4U1G | 94.2 |
| FR L for human germline grafted Ab | 6UOE | 93.6 |
| FR H for human germline grafted Ab | 6UOE | 94.6 |
| CDR L1 | 6WEQ | 91.7 |
| CDR L2 | 7NWL | 88.6 |
| CDR L3 | 7PI7 | 60.0 |
| CDR H1 | 5TR8 | 89.7 |
| CDR H2 | 1F4X | 65.9 |
| CDR H3 | 7KGV | 58.4 |

**Supplementary Table 2.** Identify the heavy chain canonical structure determining residues with MOE

| **Residue** | **Position** | **Type 1** | **Type 2** | **Type 3** |
| --- | --- | --- | --- | --- |
| E1 | HFR1 | 0 | 0 | 1 |
| V2 | HFR1 | 0 | 1 | 1 |
| L4 | HFR1 | 0 | 1 | 1 |
| E6 | HFR1 | 0 | 0 | 1 |
| G8 | HFR1 | 0 | 0 | 1 |
| G9 | HFR1 | 0 | 0 | 1 |
| L11 | HFR1 | 0 | 0 | 1 |
| V12 | HFR1 | 0 | 0 | 1 |
| K13 | HFR1 | 0 | 0 | 1 |
| C22 | HFR1 | 0 | 1 | 0 |
| F27 | HFR1 | 0 | 1 | 0 |
| F29 | HFR1 | 0 | 1 | 0 |
| S30 | HFR1 | 0 | 1 | 1 |
| W36 | HFR2 | 0 | 1 | 0 |
| V37 | HFR2 | 1 | 1 | 1 |
| R38 | HFR2 | 0 | 1 | 1 |
| Q39 | HFR2 | 1 | 0 | 1 |
| T40 | HFR2 | 0 | 0 | 1 |
| R44 | HFR2 | 0 | 0 | 1 |
| L45 | HFR2 | 1 | 0 | 0 |
| W47 | HFR2 | 1 | 1 | 1 |
| V48 | HFR2 | 0 | 1 | 0 |
| A49 | HFR2 | 0 | 1 | 1 |
| R67 | HFR3 | 0 | 1 | 1 |
| F68 | HFR3 | 0 | 1 | 1 |
| V70 | HFR3 | 0 | 1 | 1 |
| R72 | HFR3 | 0 | 1 | 1 |
| N74 | HFR3 | 0 | 0 | 1 |
| E77 | HFR3 | 0 | 1 | 0 |
| L79 | HFR3 | 0 | 1 | 0 |
| Y80 | HFR3 | 0 | 0 | 1 |
| L81 | HFR3 | 0 | 1 | 1 |
| M83 | HFR3 | 0 | 0 | 1 |
| L86 | HFR3 | 0 | 0 | 1 |
| K87 | HFR3 | 0 | 0 | 1 |
| S88 | HFR3 | 0 | 0 | 1 |
| D90 | HFR3 | 0 | 1 | 1 |
| A92 | HFR3 | 0 | 0 | 1 |
| Y94 | HFR3 | 0 | 1 | 1 |
| Y95 | HFR3 | 1 | 1 | 1 |
| C96 | HFR3 | 0 | 1 | 0 |
| A97 | HFR3 | 0 | 1 | 1 |
| R98 | HFR3 | 0 | 1 | 1 |
| W109 | HFR4 | 1 | 1 | 0 |
| G110 | HFR4 | 1 | 1 | 0 |
| G112 | HFR4 | 1 | 0 | 0 |

**Supplementary Table 3.** Identify the light chain canonical structure determining residues with MOE

| **Residue** | **Position** | **Type 1** | **Type 2** | **Type 3** |
| --- | --- | --- | --- | --- |
| I2 | LFR1 | 0 | 1 | 0 |
| L4 | LFR1 | 0 | 1 | 0 |
| Q6 | LFR1 | 0 | 1 | 0 |
| S22 | LFR1 | 0 | 1 | 0 |
| C23 | LFR1 | 0 | 1 | 0 |
| W39 | LFR2 | 0 | 1 | 0 |
| Y40 | LFR2 | 1 | 1 | 0 |
| Q42 | LFR2 | 1 | 0 | 0 |
| P47 | LFR2 | 1 | 0 | 1 |
| P48 | LFR2 | 1 | 1 | 0 |
| K49 | LFR2 | 0 | 1 | 0 |
| L50 | LFR2 | 1 | 1 | 0 |
| L51 | LFR2 | 0 | 1 | 1 |
| I52 | LFR2 | 0 | 1 | 0 |
| I62 | LFR3 | 0 | 1 | 0 |
| R65 | LFR3 | 0 | 1 | 0 |
| F66 | LFR3 | 0 | 1 | 0 |
| G68 | LFR3 | 0 | 1 | 0 |
| G72 | LFR3 | 0 | 1 | 0 |
| F75 | LFR3 | 0 | 1 | 0 |
| T76 | LFR3 | 0 | 1 | 0 |
| T89 | LFR3 | 1 | 0 | 0 |
| Y91 | LFR3 | 1 | 1 | 0 |
| C92 | LFR3 | 0 | 1 | 0 |
| F101 | LFR4 | 1 | 1 | 0 |
| G102 | LFR4 | 1 | 1 | 0 |

**Supplementary Table 4**: Comparison of RMSD values for structural components between MOE and two additional modelling programs

| Comparison section | RMSD |
| --- | --- |
| Overall structure comparison between MOE and ABodyBuilder | 0.480 |
| C-alpha between MOE and ABodyBuilder | 0.480 |
| beta-sheet between MOE and ABodyBuilder | 0.343 |
| L-CDR1 between MOE and ABodyBuilder | 0.660 |
| L-CDR2 between MOE and ABodyBuilder | 0.155 |
| L-CDR3 between MOE and ABodyBuilder | 0.902 |
| H-CDR1 between MOE and ABodyBuilder | 0.305 |
| H-CDR2 between MOE and ABodyBuilder | 0.824 |
| H-CDR3 between MOE and ABodyBuilder | 1.009 |
| Remaining Fv of LC between MOE and ABodyBuilder | 0.545 |
| Remaining Fv of HC between MOE and ABodyBuilder | 0.373 |
| Overall structure comparison between MOE and ImmuneBuilder | 0.393 |
| C-alpha between MOE and ImmuneBuilder | 0.393 |
| beta-sheet between MOE and ImmuneBuilder | 0.316 |
| L-CDR1 between MOE and ImmuneBuilder | 0.535 |
| L-CDR2 between MOE and ImmuneBuilder | 0.222 |
| L-CDR3 between MOE and ImmuneBuilder | 0.429 |
| H-CDR1 between MOE and ImmuneBuilder | 0.179 |
| H-CDR2 between MOE and ImmuneBuilder | 0.692 |
| H-CDR3 between MOE and ImmuneBuilder | 1.286 |
| Remaining Fv of LC between MOE and ImmuneBuilder | 0.489 |
| Remaining Fv of HC between MOE and ImmuneBuilder | 0.363 |

**Supplementary Table 5.** Melting temperature of humanized antibodies.

| **Molecule** | **Fc *Tm* (℃)** | **Fab *Tm* (℃)** |
| --- | --- | --- |
| BS#3 | 67 | / |
| BS#5 | 67 | / |
| BS#11 | 67 | / |
| BS#31 | 67 | / |
| T1 | 68.2 | 78.7 |
| T2 | 68.2 | 75.1 |
| T3 | 68.2 | 79.9 |
| T4 | 68.2 | 73.9 |
| T5 | 68.2 | 80.8 |
| T6 | 68.2 | 79.6 |
| T7 | 68.2 | 79.6 |
| T8 | 68.2 | 76.3 |
| T9 | 68.2 | 77.5 |
| XM Ch Ab | 68.2 | 76 |
| Pembrolizumab | 64.9 | 71 |

**Supplementary Table 6.** Comparison of type 1 canonical structure determining residues from MOE, ABodyBuilder and ImmuneBuilder models

| **Items** | **MOE vs ABodyBuilder** | **MOE vs ImmuneBuilder** |
| --- | --- | --- |
| Identical type1 residues in the heavy chain | V37, Q39, L45, W47, Y95, W109, G110 | V37, Q39, L45, W47, Y95, W109, G110, G112 |
| Different type1 residues in the heavy chain | G112(not in ABodyBuilder, but in MOE) | E46(not in MOE, but in ImmuneBuilder) |
| Identical type1 residues in the light chain | Y40, Q42, P47, P48, L50, Y91, F101 | Y40, Q42, P47, P48, L50, T89, Y91, F101, G102 |
| Different type1 residues in the light chain | T89, G102(not in ABodyBuilder, but in MOE) | I52, Y53(not in MOE, but in ImmuneBuilder) |

**Supplementary Table 7.** Interactions from MOE predicted type 1 residues in MOE, ABodyBuilder and ImmuneBuilder structure models

| **Residue** | **Interaction in MOE model** | | **Interaction in ABodyBuilder model** | | **Interaction in ImmuneBuilder model** | |
| --- | --- | --- | --- | --- | --- | --- |
| V37 (H) | HB: W36(H), R38(H), Y95(H)  HC: W36(H), L45(H), V48(H), F101(L), M106(H), W109(H) | | HB: W36(H), R38(H), Y95(H)  HC: W36(H), L45(H), W47(H), F100(L), M106(H), W109(H) | | HB: W36(H), R38(H), Y95(H)  HC: W36(H), L45(H), W47(H), V48(H), F100(L), M106(H), W109(H) | |
| Q39 (H) | HB: R38(H), T40(H), Q42(L), K43(H), Y91(L), M93(H) | | HB: R38(H), T40(H), Q42(L), K43(H), M93(H), Y95(H) | | HB: R38(H), T40(H), Q42(L), M93(H), Y95(H) | |
| L45 (H) | HB: K43(H), R44(H), E46(H)  HC: V37(H), F101(L) | | HB: R44(H), E46(H)  HC: V37(H), F100(L) | | HB: R44(H), E46(H)  HC: V37(H), F100(L), W109(H) | |
| W47 (H) | HB: E46(H), V48(H), A49(H)  HC: W36(H), V48(H), F50(H), F101(L)  PP: F50(H) | | HB: S35(H), E46(H), V48(H), A49(H)  HC: W36(H), V37(H), V48(H), F50(H), F100(L), M106(H)  PP: F50(H) | | HB: S35(H), E46(H), V48(H), A49(H)  HC: W36(H), V37(H), V48(H), F50(H), F100(L), M106(H) | |
| Y95 (H) | HB: V37(H), Q46(L), Y94(H), C96(H)  PP: W109(H) | | HB: V37(H), Q39(H), Q46(L), Y94(H), C96(H)  PP: W109(H) | | HB: V37(H), Q39(H), Q42(L), Y94(H), C96(H)  PP: W109(H) | |
| W109 (H) | HB: P48(L), M106(H), Y108(H), G110(H)  HC: L4(H), V37(H), F101(L), M106(H)  PP: Y95(H) | | HB: M106(H), Y108(H), G110(H)  HC: L4(H), V37(H), F100(L), M106(H)  PP: Y95(H) | | HB: M106(H), Y108(H), G110(H)  HC: L4(H), V37(H), L45(H), F100(L), M106(H)  PP: Y95(H) | |
| G110 (H) | HB: C96(H), W109(H), Q111(H) | | HB: C96(H), W109(H), Q111(H), G112(H) | | HB: C96(H), W109(H), Q111(H), G112(H) | |
| G112 (H) | HB: E6(H), Q111(H), T113(H) | | HB: E6(H), G110(H), Q111(H), T113(H) | | HB: E6(H), G110(H), Q111(H), T113(H) | |
| Y40 (L) | HB: W39(L), Q41(L), Y91(L), M106(H)  PP: Y91(L) | | HB: W39(L), Q41(L), Y91(L), Q93(L), M106(H)  PP: Y91(L) | | HB: W39(L), Q41(L), Y91(L), Q93(L), M106(H)  PP: Y91(L) | |
| Q42 (L) | HB: Q39(H), Q41(L), R43(L), Q46(L), T89(L), Y91(L) | | HB: Q39(H), Q41(L), R43(L), Q46(L), T89(L) | | HB: Q39(H), Q41(L), R43(L), Q46(L), T89(L), Y95(H) | |
| P47 (L) | HB: Q46(L), P48(L), K49(L) | | HB: Q46(L), P48(L) | | HB: Q46(L), P48(L) | |
| P48 (L) | HB: P47(L), K49(L), L50(L), W109(H) | | HB: P47(L), K49(L) | | HB: P47(L), K49(L) | |
| L50 (L) | HB: P48(L), K49(L), L51(L)  HC: W39(L), L51(L), I52(L), I62(L), M106(H) | | HB: K49(L), L51(L)  HC: W39(L), L51(L), I52(L), I62(L), M106(H) | | HB: K49(L), L51(L)  HC: W39(L), L51(L), I52(L), I62(L), M106(H) | |
| T89 (L) | HB: Q42(L), A88(L), Y90(L) | | HB: Q42(L), A88(L), Y90(L) | | HB: Q42(L), A88(L), Y90(L) | |
| Y91 (L) | HB: Q39(H), Y40(L), Q42(L), Y90(L), C92(L)  PP: Y40(L), F101(L) | | HB: Y40(L), K43(H), Y90(L), C92(L)  PP: Y40(L), F100(L) | | HB: Y40(L), K43(H), Y90(L), C92(L)  PP: Y40(L), F100(L) | |
| F101 (L) | HB: R44(H), T100(L), G102(L)  HC: L4(L), V37(H), L45(H), W47(H), M106(H), W109(H)  PP: Y91(L) | | HB: Q6(L), C92(L), F100(L), G102(L), G103(L) | | HB: C92(L), F100(L), G102(L), G103(L) | |
| G102 (L) | HB: R44(H), C92(L), F101(L), G103(L), G104(L) | | HB: Q6(L), G101(L), G103(L) | | HB: G101(L), G103(L) | |
|  |  |  | |  | |  |

HB: Hydrogen bond, HC: Hydrophobic contact, SB: Salt bridge, PP: pi-pi stacking


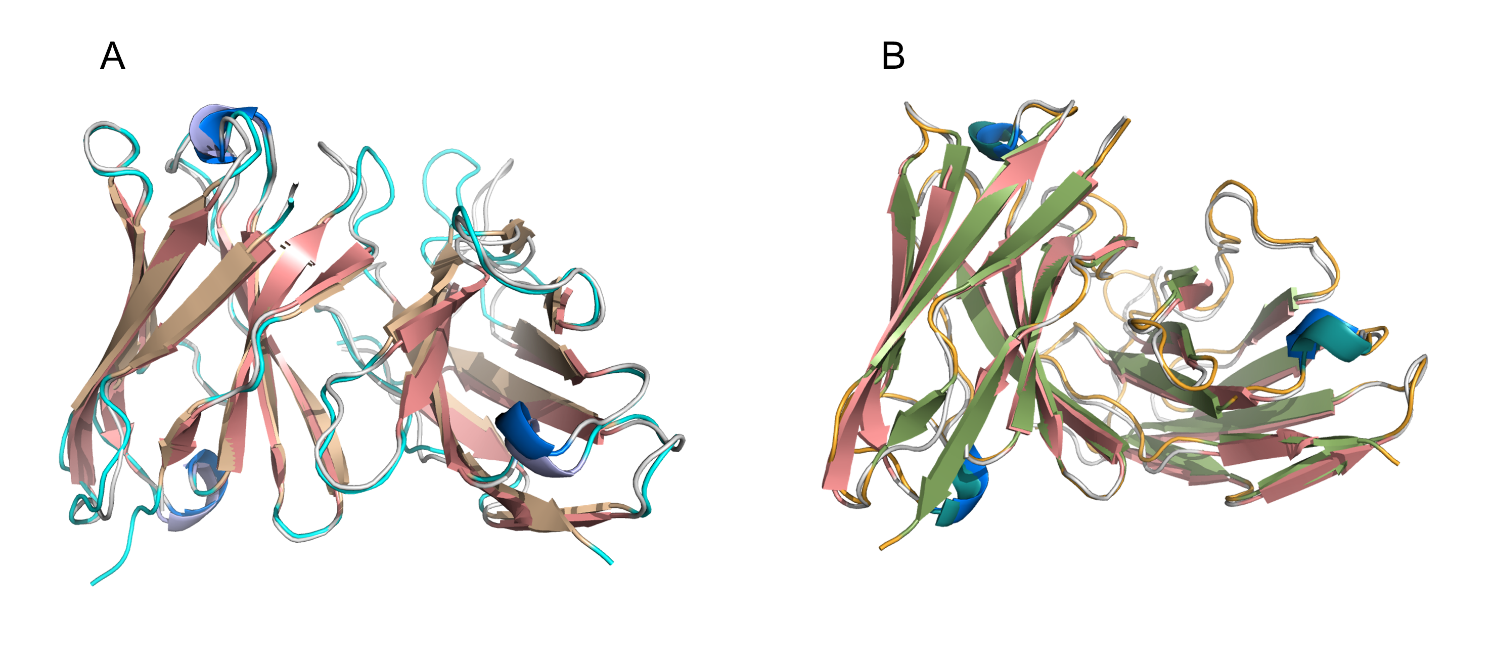


**Supplementary Figure 1.** The structures comparison between MOE, ABodyBuilder and ImmuneBuilder modeling.

(A)Comparison between MOE & ABodyBuilder. (B)Comparison between MOE & ImmuneBuilder.

MOE β: salmon; ABodyBuilder β: wheat; ImmuneBuilder β:smudge.

MOE Cα: marine; ABodyBuilder Cα: light blue; ImmuneBuilder Cα:deep teal .

MOE: gray; ABodyBuilder: cyran; ImmuneBuilder: light orange.


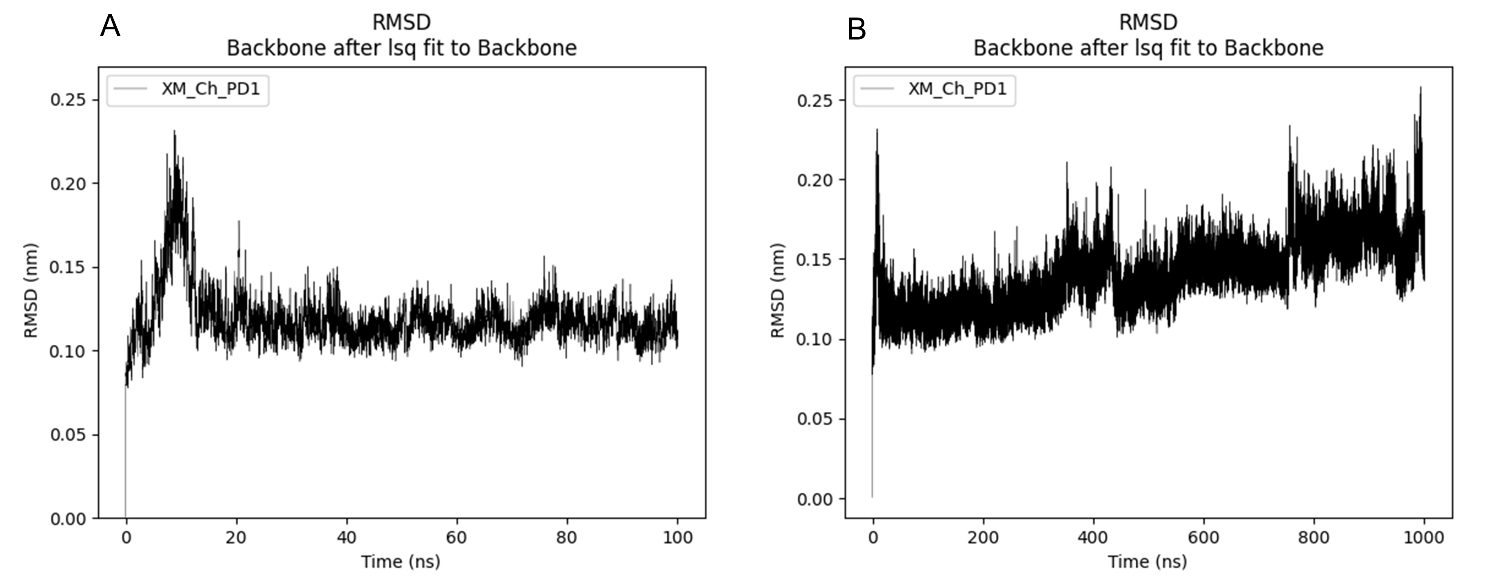


**Supplementary Figure 2.** Root Mean Square Deviation (RMSD) results of molecular dynamics simulation for XM Ch PD1, (**A**) 100 ns and (**B**) 1 µs.


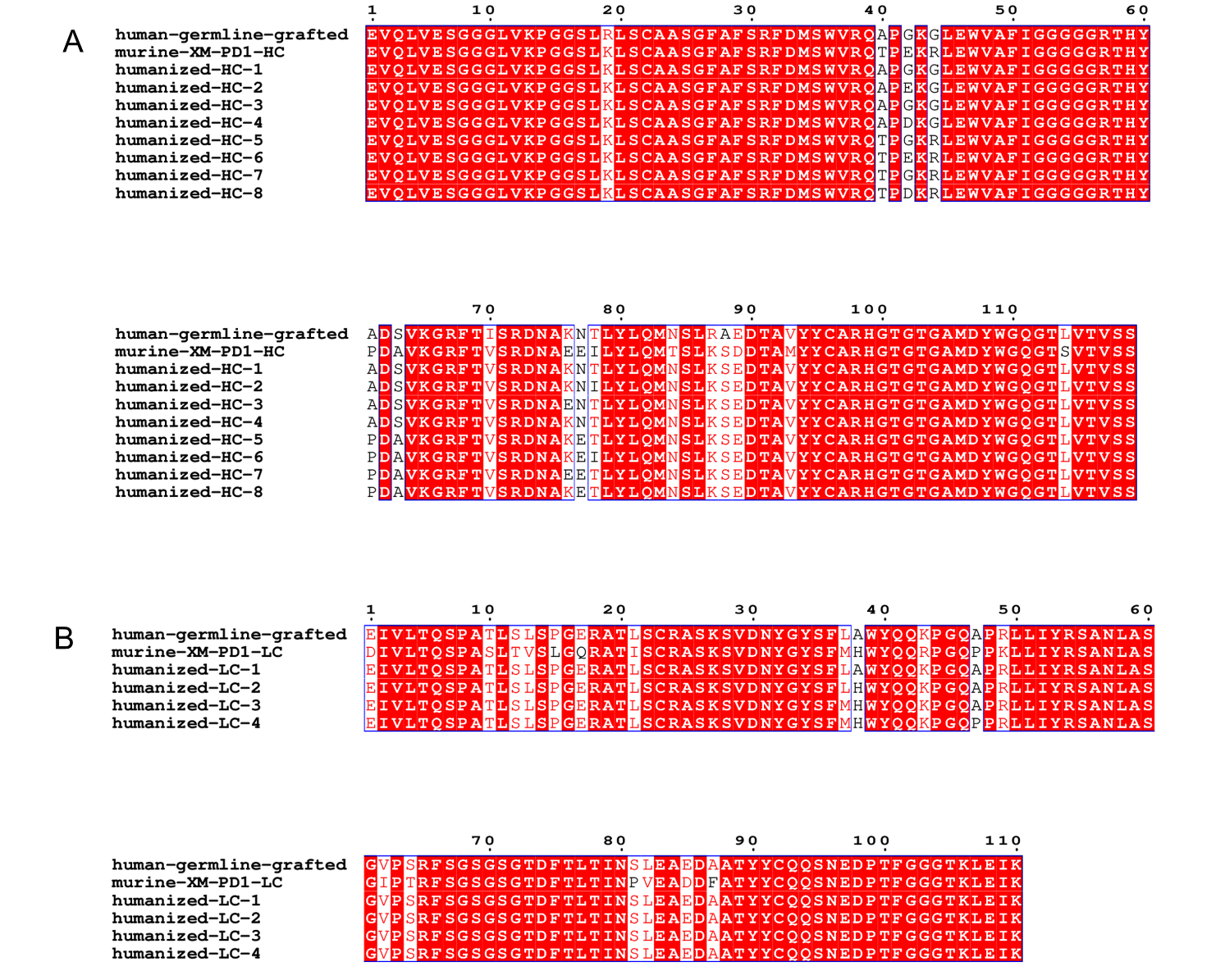


**Supplementary Figure 3.** The CDR grafting mutated list for heavy and light chains.


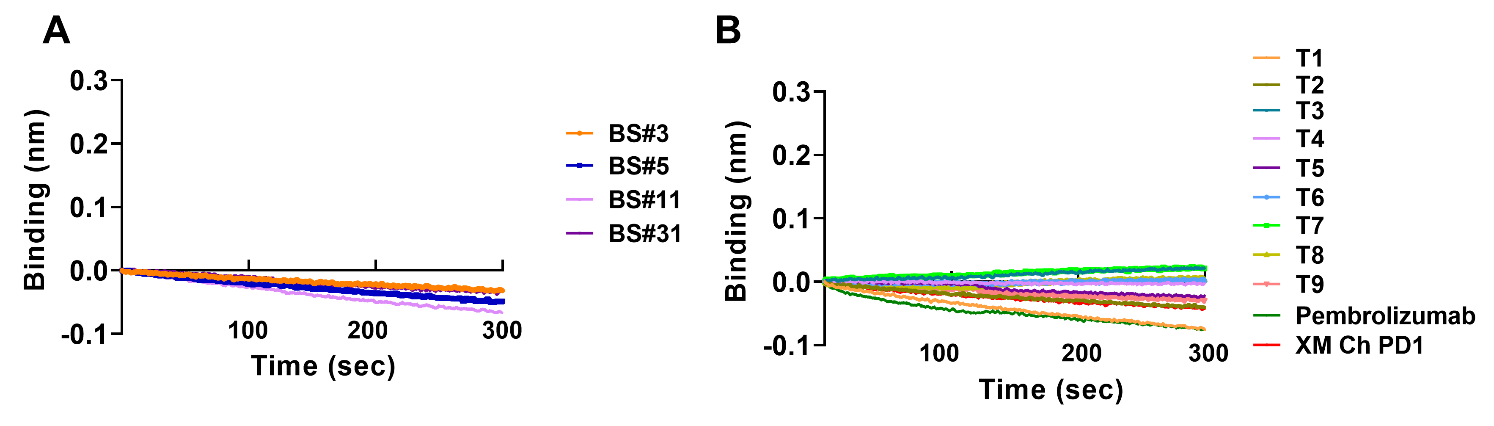


**Supplementary Figure 4.** The self-binding test using BLI. Molecules generated by CDR grafting **(A)** and framework shuffling **(B)** are shown.
